# Case Report: Non-traumatic Unilateral Forelimb Arterial Thrombosis Associated With Hyperadrenocorticism in a Dog

**DOI:** 10.3389/fvets.2021.795928

**Published:** 2021-11-25

**Authors:** Tae-Yoon Eom, Ju-Won Choi, Kyong-Ah Yoon, Soon-Wuk Jeong, Jung-Hyun Kim

**Affiliations:** ^1^Department of Veterinary Surgery, College of Veterinary Medicine, Konkuk University, Seoul, South Korea; ^2^Department of Veterinary Internal Medicine, College of Veterinary Medicine, Konkuk University, Seoul, South Korea; ^3^Department of Veterinary Biochemistry, College of Veterinary Medicine, Konkuk University, Seoul, South Korea

**Keywords:** arterial thrombosis, dog, forelimb ataxia, hyperadrenocorticism, non-traumatic, thromboelastography, tissue plasminogen activator

## Abstract

A 16-year-old spayed female Pomeranian dog was presented to the hospital with an acute onset of pain and non-weight-bearing lameness in the right forelimb. On physical examination, knuckling, coolness, pain, and cyanosis were observed in the affected forelimb. Peripheral blood glucose concentration and body surface temperature differed between the right and left forelimbs. Hypercoagulable thromboelastographic results and increased D-dimer levels were suggestive of thrombus. Accordingly, recombinant tissue plasminogen activator (rtPA) was administered intravenously. Prompt clinical improvements (including restored warmth of the affected limb) occurred, and rtPA was discontinued after two shots administered 2 h apart owing to concerns of bleeding side effects. The dog was discharged 6 days after admission, and outpatient treatment with clopidogrel was continued for the prevention of re-thrombosis. Following patient stabilization, further examinations for underlying diseases of hypercoagulability were conducted; hyperadrenocorticism (HAC) was diagnosed, and oral trilostane therapy was thus administered. Eight weeks later, the patient regained normal mobility. Finally, in the present canine patient with arterial thrombosis, thrombolysis with rtPA successfully improved clinical symptoms and the following administration of clopidogrel inhibited the formation of additional thrombus.

## Introduction

Arterial thrombosis (AT), a life-threatening disease, is common in cats but occurs very rarely in dogs (1–9). Generally, AT occurs in the hindlimbs, and very few feline cases have been reported in the forelimbs (1, 4). In dogs, there has only been a single case reported where thrombosis occurred in the forelimb associated with vehicular trauma (10). In acute AT, clinical symptoms tend to be more serious, and the prognosis is poor (2, 11, 12). Feline AT usually causes acute dramatic symptoms, such as severe limb paralysis, tachypnea, vocalization, hypothermia, azotemia, and metabolic acidosis. However, canine AT is often chronic and presents with relatively mild clinical signs, including mild locomotor abnormality, neurological deficits, and physical intolerance (11).

Canine AT has been reported to be highly influenced by many underlying diseases that promote hypercoagulability, including hyperadrenocorticism (HAC), protein-losing nephropathy (PLN), tumor, cardiac disease, hepatic disease, systemic inflammation, and immune-mediated hemolytic anemia (IMHA) (8, 10, 13–15).

To our knowledge, spontaneous unilateral forelimb AT without accidental trauma has not been reported in canine patients. Here, we describe a case of non-traumatic unilateral forelimb AT associated with HAC in a dog. In this case, recombinant tissue plasminogen activator (rtPA) was selected to dissolve the thrombi, and thromboelastography (TEG) was used before, during, and after the treatment process to detect abnormalities in the coagulation process, predict recurrence of thrombosis, and determine treatment direction.

## Case Description

**Figure 1:**
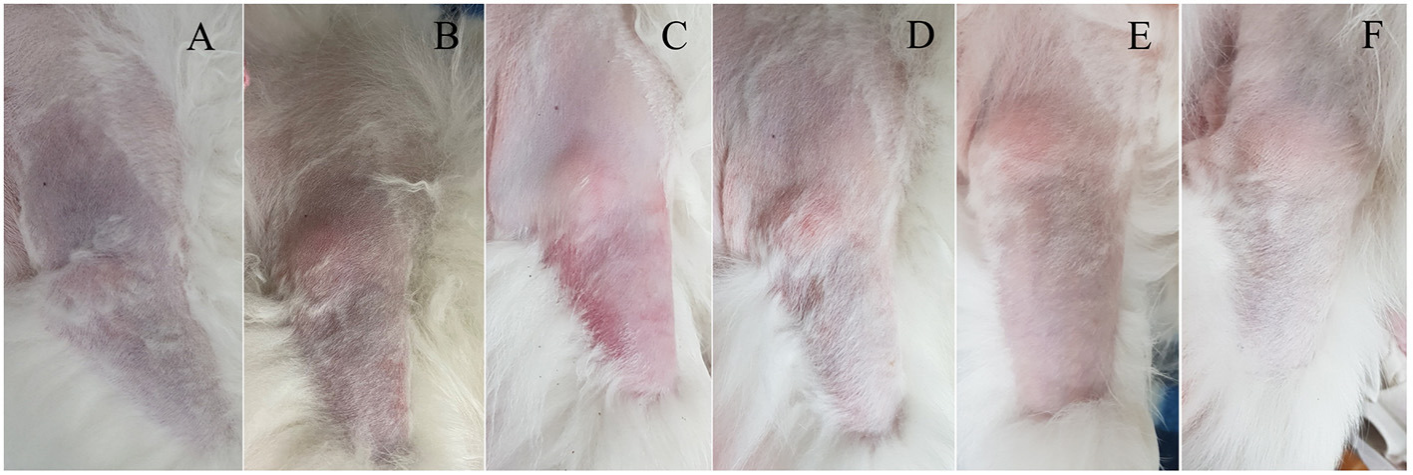
Clinical course of unilateral cyanotic forelimb with suspected arterial thrombosis in a dog following thrombolytic therapy. **(A)** Cyanosis was confirmed from the proximal third of the right forelimb on the presentation day, and the skin color became normal over time **(B–F)**. The right forelimb skin color on the following days: day 0 **(A)**, day 1 **(B)**, day 2 **(C)**, day 4 **(D)**, day 6 **(E)**, and day 21 **(F)**.

A 16-year-old spayed female Pomeranian dog was referred to Konkuk University Veterinary Medical Teaching Hospital (KU-VMTH) for acute onset non-weight-bearing lameness with severe pain of the right forelimb. The dog was initially evaluated at a local animal hospital; the referring veterinarian prescribed analgesic without tests and referred the dog to KU-VMTH. Upon arrival at the hospital, the dog showed lethargy and right forelimb weakness. Owners reported that the dog was healthy, except for regular veterinary check-ups for chronic kidney disease (CKD). On physical examination, a heart murmur was not detected, and rectal temperature was slightly low (37.4 °C). Compared with the left forelimb, the right forelimb was cool on palpation. In addition, cyanosis was identified on the proximal third of the right forelimb (Figure 1A). Neurological tests showed knuckling and non-weight-bearing lameness of the right forelimb. Severe pain was elicited even upon slight movements of the affected limb; however, there was no cervical pain. Radiographic and orthopedic examinations revealed no abnormalities.

Blood glucose concentrations, which were measured from the cephalic veins of each forelimb, showed different values between the left and right sides (left vs. right, 73 vs. 22 mg/dL; reference interval [RI]: 70–143 mg/dL). Complete blood count was unremarkable (Packed cell volume [PCV], 47.8%; platelet count, 447,000 platelets/μL; hemoglobin [Hb], 18.1 g/dL). Serum biochemical analysis revealed elevated levels of blood urine nitrogen (BUN) (105 mg/dL; RI: 7–27 mg/dL), creatinine (6.2 mg/dL; RI: 0.5–1.8 mg/dL), aspartate aminotransferase (AST) (1,549 U/L; RI: 0–50 U/L), and creatine kinase (CK) (exceeded value; RI: 10–200 U/L). D-dimer levels were markedly increased (1,217.20 ng/mL; RI: 50–250 ng/mL). TEG analysis revealed the platelet hypercoagulable state as follows: reaction (R) time = 1.2 (RI: 1.8–8.6 min), clot formation (K) time = 0.8 (RI: 1.3–5.7 min), angle (α) = 78.6 (RI: 36.9–74.6 degree), maximum amplitude (MA) = 74 (RI: 42.9–67.9 mm), and G = 14.2 (RI: 3.2–7.2 kd/s) (Table 1) (16). The ultrasonographic examination was carried out from the axillary artery to the branched brachial artery; however, the exact occlusion site was not identified. The owner refused computed tomography for diagnosis because of the anesthesia risk.

**Table 1:** Serial thromboelastographic and D-dimer results in a dog with unilateral forelimb arterial thrombosis following thrombolytic therapy.

**Variables**		**Day 0**	**Day 1**	**Day 2**	**Day 3**	**Day 6**	**Day 12**	**Day 61**	**References (16)**
TEG	R time	1.2	1.9	2.7	3.4	3.3	3.8	4.4	1.8–8.6 min
	K min	0.8	1.4	0.8	1	0.8	1	1.2	1.3–5.7 min
	Angle	78.6	71.5	77.9	75.9	79.5	75.7	72.5	36.9–74.6°
	MA	74	41.8	71.2	74.5	78.2	74.4	73.7	42.9–67.9 mm
	G value	14.2	3.6	12.4	14.6	18	14.5	14	3.2–7.2 kd/s
	Coagulation state	PHC, FHC	HypoC	PHC	PHC	PHC	PHC	PHC	-
D-dimer		1217.2	7107.25	ND	ND	1348.87	2239.38	under	50–250 ng/mL

*TEG, thromboelastography; ND, not determined; PHC, platelet hypercoagulability; FHC, factor hypercoagulability; HypoC, Hypocoagulability; R, reaction time; K, clot formation time; MA, maximum amplitude*.

## Diagnostic Assessment and Therapeutic Intervention

Based on these diagnostic tests mentioned above, AT was strongly suspected, and thrombolytic therapy was agreed upon. One and a half days after the first symptom onset, rtPA (Actilyse, Boehringer Ingelheim, Germany) therapy was started with an initial dose of a 0.2 mg/kg bolus, then 0.7 mg/kg over 30 min followed by 0.5 mg/kg intravenously over 1 h. To prevent hyperkalemia due to reperfusion injury, continuous infusion of normal saline (0.9% Normal Saline Inj., HK inno.N Corp., Seoul, Korea; 5 ml/kg/h) was performed, and 5% dextrose and sodium chloride (5% DS, HK inno.N Corp., Seoul, Korea; 2.5 ml/kg/h) were concurrently administered.

**Figure 2:**
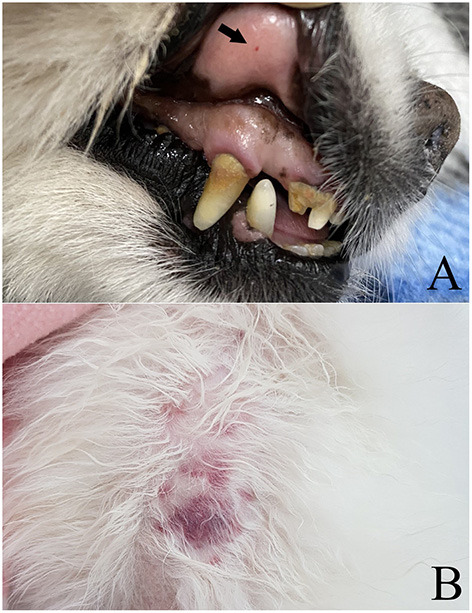
Gross findings of **(A)** petechiae of the oral mucosa (arrow) and **(B)** ecchymosis of the trunk in a dog following rtPA administration.

About an hour after the first administration, the right forelimb temperature raised on palpation, and the forelimb began swelling. The patient's pain was relieved compared with that before the administration of rtPA; the dog's whining subsided and the respiratory rate decreased to normal range. There were no bleedings on physical examination and radiography. Two hours after completion of the first administration, additional rtPA (0.8 mg/kg) was administered over 30 min. Immediately after the second dose, complete blood count testing showed decreased PCV (38.5%) and Hb (14.6 g/dL) levels. Subcutaneous bleeding on the back of the trunk and one spot hemorrhage of the oral mucosa were observed (Figure 2). Pulmonary bleeding was not confirmed by chest radiography at this time. There was no recovery of leg motility; however, the pain response was remarkably relieved, and the right forelimb temperature increased. Additional rtPA administration was not performed due to concerns about additional bleeding side effects.

The day after the presentation, additional examinations were performed to determine further treatment by identifying the cause of hypercoagulability and preventing additional thrombus formation. Abdominal ultrasound examination confirmed hypertrophy of both adrenal glands (left: cranial border, 6.9 mm; caudal border 11.1 mm. right: cranial border, 5.6 mm; caudal border, 6.5 mm). The D-dimer level was significantly increased (7,107.25 ng/mL); however, TEG revealed a hypocoagulable state (R = 1.9, K = 1.4, α = 71.5, MA = 41.8, and G = 3.6). Two days after rtPA administration, the patient's signs of pain subsided, and the condition stabilized. The adrenocorticotropic hormone (ACTH) test was then performed to determine the cause of the underlying disease. Pre-cortisol concentration was >10 μg/dL (RI: 0.5–10), and post-cortisol concentration was 23.4 μg/dL (RI: 6–18) after ACTH. HAC, in combination with clinical symptoms such as abdominal distension and hair loss, was diagnosed. Accordingly, trilostane (Vetoryl, Dales Pharmaceuticals Limited., UK; 1 mg/kg, PO, q 12 h) was prescribed for treatment. Further TEG test showed the following results: R = 2.7, K = 0.8, α = 77.9, MA = 71.2, and G value = 12.4. The administration of a platelet inhibitor was indicated. Thus, clopidogrel bisulfate (Pravic, Sinil Pharmaceutical Co. Ltd., Seoul, Korea) with an initial dose of 10 mg/kg was administered orally, followed by a subsequent daily dose of 4 mg/kg, which was maintained for the prevention of re-thrombosis.

The dog could move the right elbow joint after 6 days of rtPA administration, but knuckling continued due to incomplete mobility of the carpal joint. The pain of the right forelimb almost disappeared, and the skin color was restored to normal (Figures 1B–E). Serum CK (171 U/L) and AST (43 U/L) levels returned to the normal ranges. TEG test revealed that the hypercoagulable state (R = 3.3, K = 0.8, α = 80.7, MA = 76.9, G = 16.7) persisted; thus, clopidogrel was administered continuously. The dog was discharged due to overall relief of symptoms and started outpatient treatment. The owner was advised to provide the dog with a carpal brace to prevent knuckling. Twelve days after rtPA administration, the dog had improved enough to be able to walk when wearing a carpal brace, but it still exhibited carpal knuckling. The ACTH stimulation test revealed that the pre-cortisol level was 3.1 ug/dL, and the post-cortisol level was 7.1 ug/dL. Thus, HAC appeared to be managed successfully. However, based on the TEG test, platelet hypercoagulability persisted (R = 3.8, K = 1, α = 75.7, MA = 74.4, and G = 14.5), and the D-dimer level increased again (2239.38 ng/mL). Therefore, clopidogrel was continuously administered.

## Follow-Up and Outcomes

Twenty-one days after rtPA administration (Figure 1F), the dog presented a continuing gait pattern of knuckling, and the D-dimer level was significantly reduced (372.44 ng/mL) compared to that in previous tests. Sixty-one days after rtPA administration, the dog's gait was clinically normal, and TEG test results were as follows: R = 4.4, K = 1.2, α = 72.5, MA = 73.7, and G = 14. However, the hypercoagulable state was retained despite the trilostane and clopidogrel medication, and D-dimer levels were kept at a reduced level. Therefore, the dog was transferred to a local animal hospital for a regular recheck. Nine months after the presentation, the dog was still alive without recurrence of thrombosis.

## Discussion

AT can occur in both dogs and cats. In cats, acute paresis is the main symptom of AT and is known to recur frequently. However, in dogs, AT often occurs chronically, and minor limb function abnormalities are often seen; acute AT has been reported to occur infrequently in dogs (1–3, 5, 7). In both dogs and cats, most cases occur in the hindlimbs. In cats, AT of the forelimb alone has been reported to occur with frequencies of 4.8% in the right forelimb and 4% in the left forelimb (1). However, in dogs, there is a case report in which AT occurred where a humerus fracture was sustained and surgically corrected (10). Our case is a case of AT only in the forelimb of a dog due to an underlying disease that caused hypercoagulation without specific trauma or surgery. It is possible that the dog developed clinical symptoms due to the occurrence of AT caused by hypercoagulation induced by HAC.

TEG can evaluate the entire coagulation system from clot formation to fibrinolysis (17). Therefore, TEG is known to be a useful diagnostic test for evaluating patients at risk for thrombosis and assessing response to treatment (2). In general, in the hypercoagulation state, R and K decrease, while MA, G value, and α increase. In our case, the initial TEG test showed typical decreases in R and K values and increases in α, MA, and G values at presentation. Notably, 12 and 18 h after rtPA administration, all values returned to within the normal range, except for MA. The finding revealed a hypocoagulable state. However, about 30 h after rtPA administration, while the R value maintained normal, the K value decreased again, and the α, MA, and G values increased. These findings indicate that the effect of rtPA weakened after 30 h.

In humans, a hypercoagulation state can be observed using TEG under conditions such as obesity, postoperative period, exercise, uremia, trauma, and tumor (17). Dogs with HAC tend to exhibit hypercoagulability, and hypercoagulation does not improve even with drug treatment for HAC(. The dog in this study was diagnosed with CKD and HAC as underlying diseases. Although proteinuria or hypoalbuminemia was not confirmed in the dog, there was hypercoagulability based on the TEG test. The hypercoagulable state was not completely improved despite HAC treatment for 2 months. Therefore, it is possible that thromboembolism was caused by HAC.

Elevated D-dimer concentration could suggest the sequential activation of thrombin and plasmin in the coagulation cascade. Increased levels of D-dimers have been reported in IMHA, tumors, severe liver disease, heart failure, sepsis, and traumas, as well as in the postoperative period (18). Notably, elevated plasma D-dimer levels after thrombolytic therapy are associated with an increased risk of recurrence of thrombosis (19). In our case, the dog's plasma D-dimer concentration increased significantly after thrombolytic therapy and then decreased, but it increased again. Thus, clopidogrel was continuously administered to prevent the recurrence of thrombosis.

There is a significant difference between systemic and affected-limb peripheral blood glucose concentrations in cats and dogs with acute AT causing paralysis. This fact has been reported to be useful for an accurate diagnosis of AT (20). In the dog in this study, blood glucose levels were assessed from both forelimbs, and a significant difference was identified between the two forelimbs. This difference in blood glucose concentrations between the two forelimbs disappeared after rtPA treatment.

In dogs with AT, the prognosis is poor, and the rate of successful recovery and discharge from the hospital is about 50% (3). Limb function recovery may occur within a few days after the first treatment. However, in most cases, improvement of the limb function occurs after 2 or more weeks (11). In a previous case report of canine AT due to a forelimb fracture, moderate weight-bearing lameness was observed 48 h after treatment, and only mild lameness was observed 5 weeks after treatment (10). In our case, the dog exhibited intermittent weight-bearing lameness with knuckling 2 days after treatment. It took 8 weeks for the dog to fully recover her normal gaits. Therefore, the dog developed acute onset of symptoms but recovered successfully after thrombolytic treatment.

Treatment of AT includes dissolving existing thrombi using thrombolytic drugs or preventing new thrombus formation using anticoagulant drugs. Thrombolytic drugs including streptokinase, urokinase, and rtPA, and anticoagulant drugs, such as heparin, hirudin, clopidogrel, and rivaroxaban, are typically used (1–3, 5, 19, 21, 22). A study showed that a combination of thrombolytic agents and antithrombic agents improved outcomes of AT (23). In the present case, however, rtPA was used alone to prevent fatal complications, such as pulmonary bleeding. Notably, some case studies suggested using rtPA with bolus injections of 1 mg/kg every 1 h for a total of 10 doses (21) or an intravenous protocol of 1.4 mg/kg over 90 min (11). Accordingly, the dog described in this study received a total intravenous dose of 1.4 mg/kg over 90 min, followed by an additional intravenous dose of 0.8 mg/kg over 30 min. However, further administration was stopped due to the occurrence of subcutaneous bleeding after the second administration. Indeed, hemorrhage is the most common side effect of thrombolysis using rtPA (21). Reperfusion hyperkalemia during clot lysis is associated with high rates of mortality (19). In this case, after the administration of rtPA, reduced PCV and Hb levels, along with oral petechiae and skin ecchymosis on the trunk, were confirmed. We speculated that reperfusion hyperkalemia could cause arrhythmia and increase the risk of sudden death. Hence, we attempted to control hyperkalemia through a high flow rate of intravenous infusion of normal saline and glucose solution. Hyperkalemia appeared immediately after rtPA administration but returned to the normal range through continuous management. Further large-scale studies are warranted to reduce the occurrence of complications, such as bleeding and reperfusion injury, according to the dose of rtPA in dogs.

Our study has several limitations. First, despite the ultrasound examination, the exact location and other aspects of the occlusion could not be determined. Therefore, it was impossible to determine whether the thrombus partially or completely occluded the blood vessel. Second, even after clinical symptoms improved with rtPA administration, it was impossible to visually examine whether the thrombus had been completely dissolved. However, in the present case, the differences in blood glucose concentrations and local temperature between the two forelimbs were detected, and the hypercoagulable state was identified using TEG; these could be tentatively diagnosed as AT-associated symptoms.

## Conclusion

Herein, we present a case of AT that occurred spontaneously in the forelimb due to underlying medical diseases in a dog without traumatic events. The motor function and sensation returned to normal over 8 weeks after rtPA treatment, and the hypercoagulable state was continuously monitored by the TEG test to effectively inhibit the occurrence of additional thrombosis throughout the administration of clopidogrel. Our findings suggest that veterinarians should consider thrombosis in dogs with non-weight-bearing lameness in a forelimb, even if there is no history of trauma. In addition, TEG should be examined before and after administration of rtPA to confirm the hypercoagulable state, and medical strategy should be adjusted accordingly.

## Data Availability Statement

The original contributions presented in the study are included in the article/supplementary material, further inquiries can be directed to the corresponding author.

## Ethics Statement

Ethical review and approval was not required for the animal study because this is a case report. Written informed consent was obtained from the owners for the participation of their animals in this study.

## Author Contributions

T-YE wrote the article and contributed to the clinical assessment, diagnosis, and treatment. J-WC contributed to clinical assessment, treatment, and follow-up of the case. K-AY and S-WJ helped to write the manuscript. J-HK supervised the clinical assessment, diagnosis, and treatment of the case and wrote the manuscript. All authors read and approved the final manuscript.

## Funding

This case study was supported by the National Research Foundation of Korea (NRF-2021R1A2C2008112 to K-AY and J-HK).

## Conflict of Interest

The authors declare that the research was conducted in the absence of any commercial or financial relationships that could be construed as a potential conflict of interest.

## Publisher's Note

All claims expressed in this article are solely those of the authors and do not necessarily represent those of their affiliated organizations, or those of the publisher, the editors and the reviewers. Any product that may be evaluated in this article, or claim that may be made by its manufacturer, is not guaranteed or endorsed by the publisher.
